# Stage-Specific Adhesion of *Leishmania* Promastigotes to Sand Fly Midguts Assessed Using an Improved Comparative Binding Assay

**DOI:** 10.1371/journal.pntd.0000816

**Published:** 2010-09-07

**Authors:** Raymond Wilson, Michelle D. Bates, Anna Dostalova, Lucie Jecna, Rod J. Dillon, Petr Volf, Paul A. Bates

**Affiliations:** 1 Liverpool School of Tropical Medicine, Liverpool, United Kingdom; 2 Department of Parasitology, Charles University, Prague, Czech Republic; 3 School of Health and Medicine, Lancaster University, Lancaster, United Kingdom; Institut Pasteur, France

## Abstract

**Background:**

The binding of *Leishmania* promastigotes to the midgut epithelium is regarded as an essential part of the life-cycle in the sand fly vector, enabling the parasites to persist beyond the initial blood meal phase and establish the infection. However, the precise nature of the promastigote stage(s) that mediate binding is not fully understood.

**Methodology/Principal Findings:**

To address this issue we have developed an *in vitro* gut binding assay in which two promastigote populations are labelled with different fluorescent dyes and compete for binding to dissected sand fly midguts. Binding of procyclic, nectomonad, leptomonad and metacyclic promastigotes of *Leishmania infantum* and *L. mexicana* to the midguts of blood-fed, female *Lutzomyia longipalpis* was investigated. The results show that procyclic and metacyclic promastigotes do not bind to the midgut epithelium in significant numbers, whereas nectomonad and leptomonad promastigotes both bind strongly and in similar numbers. The assay was then used to compare the binding of a range of different parasite species (*L. infantum*, *L. mexicana*, *L. braziliensis*, *L. major*, *L. tropica*) to guts dissected from various sand flies (*Lu. longipalpis*, *Phlebotomus papatasi, P. sergenti*). The results of these comparisons were in many cases in line with expectations, the natural parasite binding most effectively to its natural vector, and no examples were found where a parasite was unable to bind to its natural vector. However, there were interesting exceptions: *L. major* and *L. tropica* being able to bind to *Lu. longipalpis* better than *L. infantum*; *L. braziliensis* was able to bind to *P. papatasi* as well as *L. major*; and significant binding of *L. major* to *P. sergenti* and *L. tropica* to *P. papatasi* was observed.

**Conclusions/Significance:**

The results demonstrate that *Leishmania* gut binding is strictly stage-dependent, is a property of those forms found in the middle phase of development (nectomonad and leptomonad forms), but is absent in the early blood meal and final stages (procyclic and metacyclic forms). Further they show that although gut binding may be necessary for parasite establishment, in several vector-parasite pairs the specificity of such *in vitro* binding alone is insufficient to explain overall vector specificity. Other significant barriers to development must exist in certain refractory *Leishmania* parasite-sand fly vector combinations. A re-appraisal of the specificity of the *Leishmania*-sand fly relationship is required.

## Author Summary

Many infectious diseases such as leishmaniasis are transmitted to people by biting insects, in this case by female sand flies. To control this and similar diseases we need to understand why particular species of sand fly transmit particular species of *Leishmania*. One important feature of the *Leishmania* parasite-sand fly interaction is the ability of the parasite to bind to the midgut wall of the fly, as it is within the gut that the parasite lives. Here we have studied the specificity of this interaction and report two main findings. The first is that only specific stages in the parasite life-cycle are capable of binding to the gut. The second is that, providing these life-cycle stages are analysed, parasite species that can be transmitted by particular sand flies are always capable of binding to their guts, but in some cases they are also capable of binding to non-transmitting sand fly species. This shows that gut binding by parasites is necessary but not sufficient to explain transmission. This research advances our understanding of *Leishmania* biology, but also shows us that there are further aspects that need to be investigated before we can fully understand the *Leishmania*-sand fly relationship.

## Introduction

Medically important protozoan parasites of the genus *Leishmania* have a two host life-cycle, alternating between various mammalian hosts and female haematophagous phlebotomine sand flies. The life-cycle within the sand fly gut is complex, varies between subgenera *Leishmania* and *Viannia*, and includes a number of distinct morphological forms given various names [1]–[5]. In the subgenus *Leishmania*, amastigote forms are released from mammalian macrophages in the midgut of the fly after ingestion of a blood meal, and transform into proliferative procyclic promastigote forms. Around 48–72 hours later, these develop into non-dividing and long nectomonad promastigotes, which escape from the peritrophic matrix-encased blood meal into the midgut lumen. Here they develop into leptomonad promastigotes (a synonym for short nectomonads [2], [3]), which enter another proliferative cycle, and by day 4 post-blood meal comprise the majority of the parasite population. The final phase is the transformation of leptomonad forms into mammalian infective stages, metacyclic promastigotes, and by 7–10 days post-blood feeding over 60% of the parasite population in the region of the stomodeal valve (junction between midgut and foregut) is comprised of this form [4], [5].

One essential event in the establishment of *Leishmania* infections in the sand fly is the attachment of the parasites to the midgut epithelium. By anchoring themselves to the midgut the parasites help to prevent their expulsion from the gut during defecation, and it has been postulated that this binding is the main determinant of parasite-vector specificity [6]–[9]. The mechanism of binding has been most intensively studied in *L. major* infections of *Phlebotomus papatasi* [6], [7], [10]–[12]. The promastigote surface glycoconjugate lipophosphoglycan (LPG) was implicated as the parasite molecule involved in midgut attachment of *L. major* [6]. In this parasite-vector combination, competitive inhibition with oligosaccharides derived from LPG [6], anti-LPG antibodies [6], and mutant strains that either lack LPG entirely [9] or produce LPG that lacks the carbohydrate side-chain moieties present on wild-type *L. major* LPG [12], all caused a significant reduction in the binding of *L. major* to *P. papatasi* midguts in vitro and/or a reduction/ablation in the parasite's ability to complete its life-cycle in the sand fly. A corresponding galactose-binding lectin or galectin (PpGalec) has been described on the midgut of *P. papatasi* that binds to *L. major* LPG, and antibodies against PpGalec inhibit promastigote attachment [13]. Similar, although less complete, analyses have been performed on various additional vector-parasite combinations, leading to the hypothesis that LPG is the key molecule for midgut attachment in all *Leishmania* species [14]. One unexplained observation is that attachment usually occurs via the flagellum, which is seen to insert between the microvilli, whereas LPG is found over the whole surface of promastigotes. The reasons may be mechanical, the flagellum being at the anterior of the cell and more easily able to fit in between the microvilli, but could indicate that other molecules have a role to play in attachment. However, in subsequent work it was shown that LPG is not essential for completion of the parasite life-cycle in all *Leishmania*/sand fly infections [15], [16]. This suggests either that attachment is not always obligatory for life-cycle progression, in contradiction to the current paradigm, or that an alternative or supplementary non-LPG mediated attachment mechanism exists.

Non-LPG mediated attachment of promastigotes to the sand fly gut has only been observed in sand flies termed “permissive vectors” [15]. This concept relates to the subdivision of vector sand fly species into two broad categories, permissive and specific/restricted/refractory vectors [17]–[19]. Specific vectors only allow the maturation and transmission of a single *Leishmania* species. For example, *P. papatasi* supports development of *L. major* only, and is refractory to all other species tested [7]. Similarly, *P. sergenti* supports the maturation of typical *L. tropica* strains [20], [21]. The majority of sand flies, however, fall into the permissive category, and multiple *Leishmania* species are able to survive and mature within the gut of such a species if given the chance by experiment or nature. Examples of permissive flies are the New World species, *Lutzomyia longipalpis* [4], [22], [23], and the Old World species *P. argentipes*, *P. arabicus, P. halepensis* and *P. perniciosus* [7], [15], [16], [21], [24], which allow the maturation of practically all *Leishmania* species tested under experimental conditions. For example, the Old World parasite *L. major* is able to complete its development in the New World vector *Lu. longipalpis* [22], which is the natural vector for *L. infantum* (syn. *chagasi*). The current evidence indicates that infections of specific vectors have a strict dependence on species-specific LPG for binding the parasites to the midgut. However, in permissive vectors the picture is less clear, LPG does not appear to be essential, and by implication there appears to be an additional LPG-independent binding mechanism in these vectors [15]. In this study, we examined midgut-binding in both specific and permissive vectors.

## Materials and Methods

### Parasites and sand flies

The following parasite isolates were used in this study: *Leishmania braziliensis* MHOM/BR/84/LTB300; *L. infantum* (syn. *chagasi*) MHOM/BR/76/M4192; *L. major* LV561 MHOM/IL/67/LRC-L137; *L. mexicana* MNYC/BZ/62/M379; and *L. tropica* SU23 MHOM/TR/98/HM. Laboratory colonies of three sand fly species were used: *Lutzomyia longipalpis* (origin from Jacobina, Brazil); *Phlebotomus papatasi* (Turkey); and *P. sergenti* (Turkey).

### Generation of *Leishmania* life-cycle stages

*Leishmania* parasites were assigned to life-cycles stages according to the classification of Rogers *et al*. [4]. *L. mexicana* lesion amastigotes were transferred into promastigote culture medium (M199 medium containing 25 µg/ml gentamicin sulphate (Sigma), 1x BME vitamins (from 100x stock, Gibco), 20% foetal bovine serum (FBS; Sigma), and 2% urine) at 26°C and promastigote cultures enriched for procyclic, nectomonad, leptomonad and metacyclic forms were collected after 1, 2, 4 and 8 days of in vitro culture, respectively [4]. For *L. infantum* it was not possible to obtain sufficient quantity of promastigotes direct from mouse spleen homogenate amastigotes. For this species, amastigotes of *L. infantum* were transferred into promastigote culture medium and counted daily until exponential growth of promastigotes was observed (2–3 days). The parasites were then passaged at a concentration of 5×10^5^/ml into fresh medium and cultured for 2–3 days or 7 days to produce leptomonad-enriched or metacyclic-enriched cultures, respectively. Procyclic and nectomonad promastigotes were derived from metacyclic cultures that had been centrifuged and resuspended in Grace's Insect Medium (GIM) containing 20% FBS, pH 5.5 and cultured at 32°C until transformation to amastigote forms had occurred (1–2 days). The resulting amastigotes were then transferred back into promastigote culture medium and cultured at 26°C for 24 hours to produce procyclic-enriched cultures or 48 hours to produce nectomonad-enriched cultures. For additional species of *Leishmania* a methodology similar to that used with *L. infantum* was employed. For species-species comparisons, leptomonad-enriched cultures were prepared for each parasite isolate.

Parasites from the enriched cultures were cryopreserved in 10% dimethylsulphoxide, 1 ml aliquots stored under liquid nitrogen, and thawed as required for binding assays. Thawed aliquots were washed twice in promastigote culture medium to remove dimethylsulphoxide prior to use. Viability was then checked microscopically and very few if any dead (non-motile) promastigotes were observed in the majority of cases. However, in rare instances where more than 5% of the promastigotes were not viable such parasites were not used in experiments. This precaution was taken in case the original population was in poor condition, rather than due to the presence of dead parasites per se, as these did not bind to midguts under the conditions of the assay. All of the populations used were enriched to at least 80% of the relevant life-cycle stage, with the exception of *L. infantum* nectomonad promastigotes, which could only be obtained to 60% purity. All procedures involving animals were performed in accordance with UK Government (Home Office) and EC regulations, and were approved by the University of Liverpool Animal Welfare Committee.

### Midgut-binding assay

*Lu. longipalpis* and *P. sergenti* midguts were dissected in GIM four days after blood-feeding. *P. papatasi* were dissected 6 days after blood-feeding due to the blood meal digestion period in this species being slightly longer. Hindgut, foregut and Malpighian tubules were removed and the midgut carefully opened longitudinally using a bevelled, fine glass needle. The needles were made from borosilicate 3·5 inch hematocrit capillary tubes (Drummond, USA) drawn into fine tips using a PC-10 Narishige Puller. Before use the needles were bevelled using an EG-44 Narishige microgrinder. Parasites were labelled for 1 hr at 26°C with either 0·04% (v/v) Syto21 Green or 0·2% Syto40 Blue fluorescent dyes (Molecular Probes), washed 3 times in M199 medium and twice in GIM, then resuspended in GIM at a concentration of 1×10^8^ cells/ml. The two parasite populations were mixed in equal quantities before overlaying 10 µl of the mixture on each gut and incubating at 26°C for 45 minutes in a humid chamber. The gut was washed 5 times by transferring to fresh drops of GIM, placed in 2 µl of cooled CyGel (Biostatus Ltd.) on a microscope slide, and carefully flattened so that no areas of the gut were folded over. The slide was warmed to solidify the CyGel, thereby fixing the gut in place, before mounting in Prolong Gold antifade reagent. Bound parasites of both populations were visualised simultaneously using an Olympus BX60 fluorescence microscope at x400 magnification with a CFP/YFP-A filter set (Semrock Inc.), the number of parasites from each population counted in one field of view per midgut, and their relative proportions determined. No consistent variation in binding along the length of the dissected guts was observed, but damaged areas were not used. Smears were made from the labelled parasites, stained with Giemsa's stain and measured to determine the proportion of each life-cycle stage in each culture. Each experiment was repeated with the colour labelling of the parasite populations switched, so that any potential dye-specific bias was negated (technical repeat), and each pair of comparisons was performed at least twice (biological repeat). The difference between the proportions of bound parasites of two populations was evaluated statistically by means of an ANOVA test using the R software (http://www.r-project.org/).

## Results and Discussion

### Development of the parasite-binding assay

**Figure 1:**
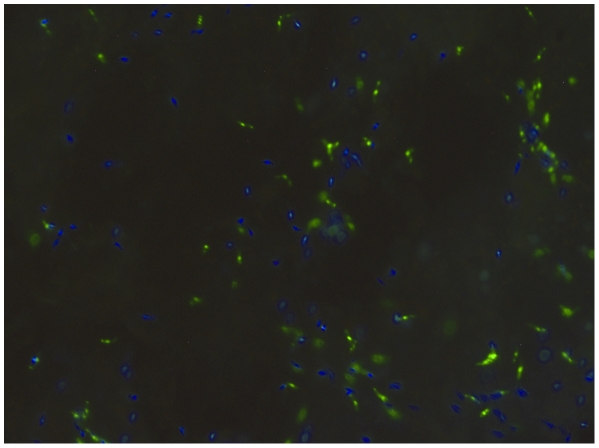
Binding of two *Leishmania* populations to a sand fly midgut. In the example shown, *L. mexicana* nectomonad promastigotes are labelled with Syto Blue and *L. mexicana* leptomonads with Syto Green, and are bound to a flattened *Lu. longipalpis* midgut. The gut was photographed after washing, and all the bound promastigotes were alive as revealed by movements of their cell bodies. Some areas with fewer promastigotes can be seen, but these had no obvious distribution.

Previous investigations of *Leishmania*-sand fly interactions have shown that, from around the time of escape from the peritrophic matrix and continuing throughout the course of the infection, a proportion of promastigote forms bind to the gut epithelium by intercalating their flagella between the microvilli and/or direct contact of the cell body [2], [25]. In this study a comparative binding assay was developed and utilised to investigate the stage- and species-specificity of gut binding. This assay differs from previous assays in several respects. First, it utilises direct microscopic examination of a mixture of two differentially stained parasite populations to individual dissected sand fly midguts (Fig. 1). This relative binding methodology was used because, even under optimum conditions, individual sand flies (and their midguts) will be in slightly different physiological states, of slightly different ages, and dissection of a fully intact gut (required for reliable absolute numbers) is also technically demanding. Considerable individual variation in the numbers of parasites bound to sand fly guts has been noted before [7], [20]. In preliminary experiments the absolute numbers of single (unmixed) populations of promastigotes binding to midguts were determined, and as expected large variations in binding to the surface midgut epithelium were noted, for example ranging from 91 to 847 per field of view (average  = 433±44·2 SEM) for *L. infantum* with *Lu. longipalpis*. It is also relevant to note previous observations that the binding of *L. major* to *P. papatasi* midguts was not spatially uniform, occurring on galectin-expressing patches of cells of varying size [13]. The protocol developed in the current study minimises the biological variability of previous studies associated with comparison of binding of different populations to different midguts.

Another feature of these experiments was that they were performed using guts dissected from flies at an appropriate time post-blood feeding with respect to *Leishmania* development. Surprisingly, all previous studies have used “sugar-fed” flies, i.e. flies that had emerged after pupation but had not yet taken a blood meal. However, blood feeding is known to exert profound physiological changes on the midgut epithelium, the secretion of digestive enzymes and production of the peritrophic matrix being two obvious examples. Thus, it is unwise to assume that binding of parasites to sugar-fed flies would accurately mimic binding in an infected sand fly following blood feeding, the analogous situation under natural conditions. This is further supported by possible upregulation of galectin expression in *P. papatasi* following blood feeding [13]. Therefore, the results in this study were all derived from assays performed with guts dissected from flies 4 (the majority) or 6 (*P. papatasi*) days after blood feeding, these being the times at which maximum binding of promastigotes has been reported to occur *in vivo*.

Finally, previous *in vitro* midgut binding assays have all been performed in phosphate buffered saline [6,7,20,26,27). In addition to being non-physiological, and as has also been noted by Kamhawi *et al*. [20], we found sand fly midguts were not very stable in this solution and their structure began to deteriorate after 10 minutes incubation at 26°C. Therefore, we performed the assays in GIM, which as a customised insect culture medium resolved this problem, with the guts maintaining their structural integrity and remaining amenable to manipulation for at least 4 hours. Moreover, GIM is also a suitable medium for the growth and maintenance of *Leishmania* promastigotes [28].

The assay described here has a number of advantages over the established *in vitro* assay [6], [29]. One of the major differences is in the method of quantification. Here, we determined the number of *Leishmania* parasites from two distinct populations that were bound to the same midgut. By expressing these numbers as a ratio of the numbers of each parasite population bound per gut, a large amount of the variation between samples was eliminated. Individual variation is reduced in the new assay by only counting parasites from one microscope field of view per gut instead of homogenising the whole gut and counting the released parasites on a haemocytometer, which, due to differences in sizes of the guts and quality of dissections, introduces a source of variation. In contrast to previously described *in vitro* assays, this one is suitable also for studies on midguts from wild-caught sand flies. Nevertheless, we strongly encourage researchers to use colonized females as they represent more standardized material.

### Life-cycle stages of *L. infantum* and *L. mexicana* involved in binding to the midgut

**Figure 2:**
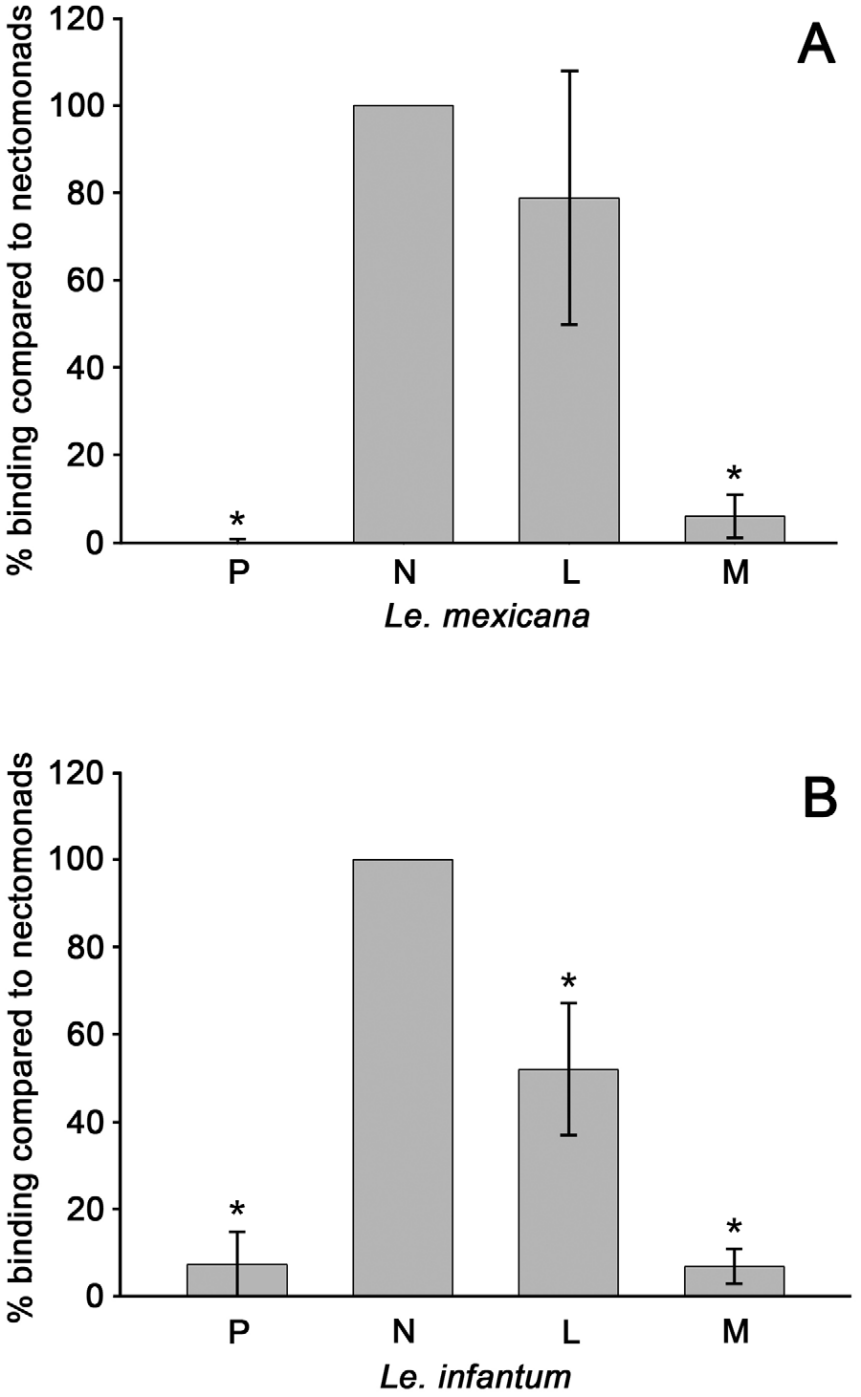
Comparative binding of enriched parasite populations to *Lu. longipalpis* midguts. All populations were compared to the binding of the nectomonad-enriched culture. (A) *L. mexicana* (11 experiments) (B) *L. infantum* (5 experiments). Cultures were enriched for: procyclic promastigotes (P); nectomonad promastigotes (N); leptomonad promastigotes (L) and metacyclic promastigotes (M). Each bar represents the mean percentage ± S.D. and the asterisks indicate a significant difference to the nectomonad population (>20 dissected guts examined per experiment).

*Leishmania* promastigotes occur in four major developmental forms in sand flies [4]: procyclic, nectomonad, leptomonad and metacyclic promastigotes. In addition there is a small population of haptomonad promastigotes and paramastigotes attached to the cuticular surfaces of the stomodeal valve and foregut by specialised hemidesmosomal structures, a completely different attachment mechanism to that being examined in this study, but these are not involved in attachment to the midgut. To determine the life-cycle stages involved in binding to the sand fly midgut, we initially attempted to measure parasites bound *in situ* to midguts of *Lu. longipalpis*. These experiments indicated that nectomonad and leptomonad promastigotes were the major forms responsible for binding to the midgut (data not shown). However, a significant technical problem in measuring such bound parasites *in situ* is that accurate measurement relies on the parasite being perfectly flat in relation to the plane of focus. Any deviation in the vertical angle of the parasite results in a shortening of the perceived length. Therefore, we adopted the comparative binding assay with *Lu. longipalpis* to determine the binding of the different developmental forms. Promastigote populations of *L. mexicana* and *L. infantum* enriched for the four different types of promastigotes were prepared and used. Comparing the binding of a population enriched for nectomonad promastigotes with a leptomonad-enriched population showed that nectomonads bound to the midgut in higher numbers in both *L. mexicana* and *L. infantum* (Fig. 2), although the difference was not statistically significant in *L. mexicana*. The leptomonad-enriched populations bound at levels 52·0±9·9% (P<0·01) and 78·7±16·3% (P = 0·97) of the nectomonad-enriched populations in *L. infantum* and *L. mexicana*, respectively. The nectomonad-enriched cultures were, therefore, used as the standard to which the other life-cycle stages were compared. In confirmation of the observations made with the direct measurements of bound parasites, there was little significant binding of either procyclic or metacyclic promastigote-enriched populations to the midgut in comparison to the nectomonad population in either *L. infantum* (procyclic 7·1±1·5%, P<0·01; metacyclic 6·5±3·7%, P<0·01) or *L. mexicana* (procyclic 0·55±0·55%, P<0·01; metacyclic 5·6±4·6%, P<0·01). Similarly, there was minimal binding of metacyclic promastigotes relative to leptomonad promastigotes. Further, direct observation indicated that virtually all of the bound parasites from the procyclic and metacyclic populations were derived from contaminating nectomonad and leptomonad promastigotes, respectively, that made up to 20% of the respective starting populations. Total numbers of bound parasites per field of view ranged from less than 5 to a few hundred depending on which combination of cell types were being examined. Thus numbers of bound procyclic and metacyclic promastigotes were on average 1–2 parasites per field, whereas averages for nectomonad and leptomonad promastigotes ranged from 50 to 200 parasites per field. Since the assay used is a comparative binding assay, it was possible the procyclic and metacyclic forms were able to bind but were being out-competed by the strongly binding nectomonad and leptomonad forms. This was, however, found not to be the case in experiments comparing the binding of procyclic versus metacyclic populations (data not shown), where neither were observed binding to the guts in significant numbers.

The results presented here provide a detailed analysis of the life-cycle stages involved in midgut-binding, with the only two forms observed binding to the midgut epithelium being the nectomonad and leptomonad promastigotes. Nectomonads are the predominant form 2–4 days post-blood feeding, while the leptomonad forms are present in high numbers from 4 days onwards [4], [5]. The main purpose of binding to the sand fly midguts is thought to be in preventing expulsion from the gut due to peristalsis, particularly during defecation of the blood meal [14], therefore, the binding data corresponds well with the promastigote forms present at the time binding to the gut is most expected. The predominant form bound to the sand fly gut at a particular point in development will be the product of various factors. From these results we can be confident that nectomonad promastigotes will constitute the first population of bound promastigotes, since these preceed leptomonad forms. However, since the detachment rate is unknown the subsequent population is difficult to predict. Attachment could be effectively permanent if the detachment rate is very low, or it could be very dynamic with promastigotes continuously attaching and detaching. In the former scenario replacement of the nectomonad population might be relatively slow even though the total gut population becomes dominated by leptomonad forms; in the latter scenario the replacement of nectomonad with leptomonad forms could occur relatively quickly. Resolution of this will likely require dissection or real time imaging of infected flies combined with stage specific markers for nectomonad and leptomonad forms, which is not currently possible but an interesting area for future investigation.

The inability of metacyclic promastigotes to bind is intuitively expected, since these are the infective forms of *Leishmania* and a propensity for strong binding to the gut lining would be a considerable disadvantage at this stage of its life-cycle. The loss in binding capacity of metacyclics has been attributed to changes in the structure of the major parasite surface molecule, LPG, during metacyclogenesis [6], [27], [29]–[31]. The LPG on the surface of metacyclics has a much longer phosphoglycan backbone and changes the nature of its terminally exposed sugar residues during metacyclogenesis. Different *Leishmania* species have different LPG modification mechanisms: *L. major* caps the terminal galactose residues with arabinose [6]; *L. donovani* and *L. infantum* exhibit conformational changes that mask the terminal sugars [26], [29]; and *L. braziliensis* adds glucose side-chains [27]. Another possible factor influencing binding in vivo is the secretion of phosphoglycans. Although LPG itself is not shed by promastigotes in vivo, filamentous proteophosphoglycan(s) are [32], and these could act as competitive inhibitors of LPG-mediated binding since they share similar phosphoglycans. This could assist in the detachment of nectomonad or leptomonad promastigotes and/or prevent the re-attachment of metacyclic promastigotes. However, changes in LPG/PPG structure appear to be insufficient by themselves to explain lack of metacyclic binding in permissive vectors where non-LPG mediated attachment mechanisms have been described.

Procyclic promastigotes, on the other hand, may be unable to bind to the midgut due to a lack of (sufficient) LPG on their surface, since *in situ* immunohistological studies in *L. major* failed to detect any surface LPG until day 3 post-blood meal [33], a time point which corresponds with the appearance of the nectomonad promastigotes. There is also the possibility of procyclic promastigotes being unable to bind to the midgut for mechanical reasons. Morphologically, procyclic promastigotes differ from nectomonad and leptomonad promastigotes in having a short flagellum [4], and, therefore, reduced mobility. Some motility appears to be essential for midgut binding, as dead non-motile promastigotes were not able to attach. The lack of binding also raises the possibility of the sand fly binding receptors being restricted to the base of the microvilli where they cannot be reached by the short procyclic flagella and/or a mechanical interaction between the flagellum and microvilli being important in initiating binding.

### Species-species binding comparisons

**Figure 3:**
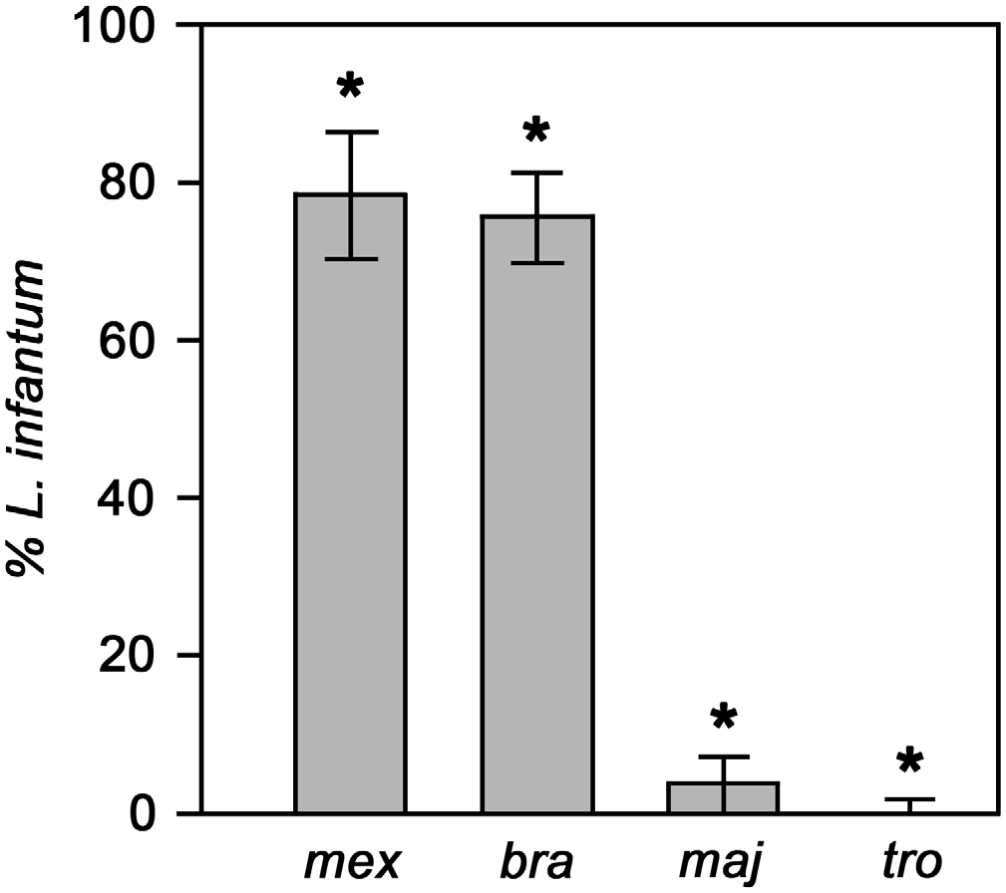
Binding of *L. infantum* to midguts from *Lu. longipalpis* in the presence of various *Leishmania* species (4 experiments). Competing species: *mex*  =  *L. mexicana*; *bra*  =  *L. braziliensis*; maj  =  *L. major*; *tro*  =  *L.tropica*. Bars represent the mean percentage contribution of *L. infantum* in the bound population ± S.D. and the asterisks indicate a significant difference to the *L. infantum* population (>20 dissected guts examined per experiment).

In the next series of experiments we assayed the binding of *L. infantum* to *Lu. longipalpis*, a permissive vector and natural host of this parasite in South America, in the presence of various other species of *Leishmania* (Fig. 3). In these experiments the results are displayed such that if both species of parasite bind equally well they will give a value of 50% on the y-axis. If *L. infantum* binds in greater relative numbers values of greater than 50% will be found; conversely poorer binding will yield values lower than 50%. The data shows that *L. infantum* is able to bind more effectively to *Lu. longipalpis* than either *L. mexicana* or *L. braziliensis* (P<0·01 in both cases), approximately 3–4 fold higher (Fig. 3). These two parasites are also found in the New World. On the other hand, *L. major* and *L. tropica* both strongly out-competed *L. infantum* in binding to *Lu. longipalpis* (Fig. 3). Binding of *L. major* accounted for over 90% of the parasites counted: not only were bound *L. major* proportionally more abundant than *L. infantum* (P<0·01), but the number of *L. infantum* bound per gut was greatly reduced (from ∼90 to ∼20 per field), indicating that *L. major* was genuinely out-competing *L. infantum* for binding sites. This effect was even more pronounced with *L. tropica*, which completely out-competed *L. infantum* binding to *Lu. longipalpis* midguts (P<0·01; ∼7 per field). Such competition could never occur in nature, as *L. major* and *L. tropica* are restricted to the Old World. However, these results show that the natural parasite is not necessarily the species that can always bind *in vitro* most efficiently to the midgut of its vector.

**Figure 4:**
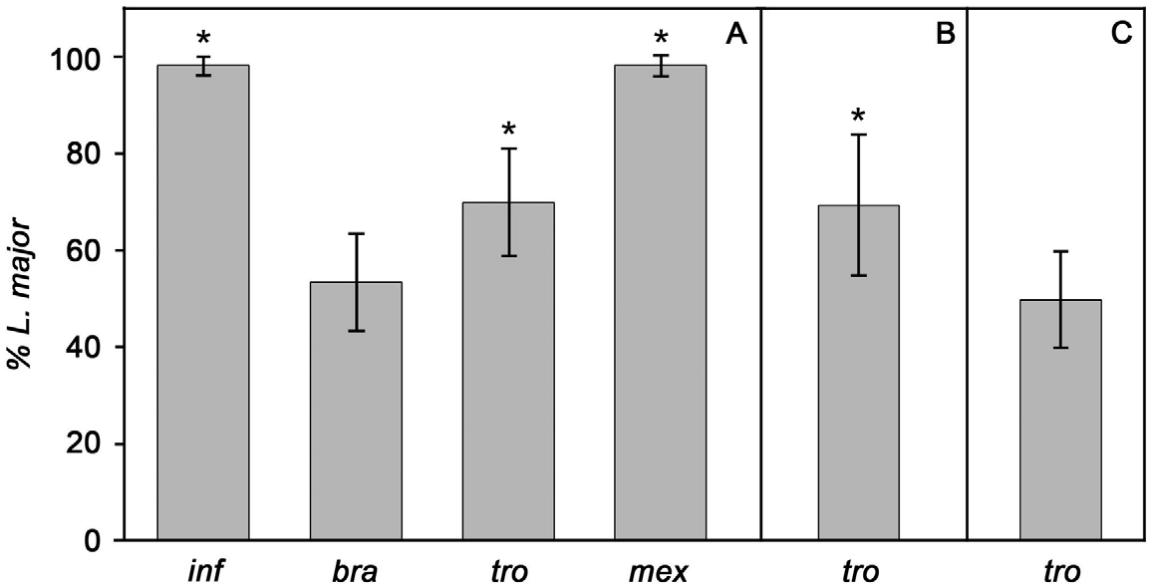
Binding of *L. major* to midguts from sand fly species in the presence of various *Leishmania* species (6 experiments). (A) Binding to *P. papatasi* midguts. (B) Binding to *Lu. longipalpis* midguts. (C) Binding to *P. sergenti* midguts. Competing species: inf  =  *L. infantum* FVI; *bra*  =  *L. braziliensis*; *tro*  =  *L. tropica*; *mex*  =  *L. mexicana*. Bars represent the mean percentage contribution of *L. major* in the bound population ± S.D. and the asterisks indicate a significant difference to the *L. major* population (>20 dissected guts examined per experiment).

In the final series of experiments we investigated the binding of *L. major* to midguts from various vectors, including to those of its natural host *P. papatasi* (Fig. 4A). Some of the results were as predicted from known vector-parasite combinations, but again with some interesting exceptions. *L. major* outcompeted *L. infantum* on *P. papatasi* (P<0·01), this result being more expected than that found with the permissive vector reported above. Similarly, *L. major* out-competed *L. mexicana* (P<0·01), which cannot complete its development in *P. papatasi*. On the other hand *L. braziliensis* was able to bind to *P. papatasi* equally well as *L. major* (P = 0·34*)*, a somewhat unexpected finding given that this vector cannot support the development of *L. braziliensis* [34], or the closely related peripylarian parasite *L. panamensis* [35]. Another intriguing result was provided by *L. tropica*, which, representing about 30% of the population in competition with *L. major*, was clearly capable of *in-vitro* binding to this specific vector to a significant degree (not as well as *L. major*, though, P<0·01). The competition between *L. major* and *L. tropica* was explored further in two additional vectors, *Lu. longipalpis* and *P. sergenti*. The results with *Lu. longipalpis* (Fig. 4B) were similar to those with *P. papatasi*, *L. major* being somewhat more efficient than *L. tropica* (P<0·01). However, the results with *P. sergenti* were again contrary to expectations (Fig. 4C). This is the specific vector of *L. tropica* and, therefore, this might be expected to out-compete all other parasites. However, there was equal *in vitro* binding of *L. major* and *L. tropica* in *P. sergenti* (P = 0·96). The absolute numbers of bound promastigotes varied in these species comparisons. The lowest and highest numbers observed were for *L. infantum* and *L. major* when competing with each other in *P. papatasi*, 2–10 parasites per field to over 1000, respectively.

The ability of different sand flies to support the growth of *Leishmania* differs from species to species. Some, such as *P. papatasi* and *P. sergenti* are highly specific, and these only allow the full development of *L. major* and *L. tropica* respectively, while others are more permissive (e.g. *Lu. longipalpis* and *P. perniciosus*) and allow the development of a number of species [19]. At each stage of its development the parasite has to overcome or evade the defences of the sand fly that may prevent progression of the life-cycle. One key barrier is the ability of *Leishmania* to attach to the midgut after escaping from the peritrophic matrix, and thus greatly enhance their chances of remaining in the gut after defecation. For *P. papatasi*, the results presented here indicate a complete inability of *L. infantum* and *L. mexicana* to attach to the midgut, suggesting midgut binding is highly likely to be a crucial step in parasite establishment in this species of sand fly. Due to the competitive nature of the binding assay used, it is, however, impossible to ignore the possibility that *L. infantum* and *L. mexicana* are able to bind weakly to the midguts but are being out-competed by the *L. major* promastigotes for binding sites. Both of these interpretations are compatible with the published literature. For example, Pimenta *et al*. [7] reported binding by *L. infantum* to *P. papatasi* midguts was approximately 10% of that observed with *L. major*.

Neither *L. braziliensis* [34] nor *L. tropica* [7] are capable of completing their life-cycles within *P. papatasi*, but both bound well to *P. papatasi* guts *in vitro*, with *L. braziliensis* showing no difference in its binding abilities to those of *L. major*. A similar observation was made by Warburg *et al.* [10] in studies on the flagellar attachment of *L. panamensis*. Obviously, in these cases the ability to bind to the midgut *in vitro* is not the selective refractory barrier to infection of these species in *P. papatasi*. Experimental infections with *L. panamensis* in *P. papatasi* [35] demonstrated the majority of flies lost their infection during defecation of the blood meal. That most flies retained an intact peritrophic matrix prior to defecation suggests the parasites were probably unable to break free of the blood meal and enter the midgut lumen, rendering their ability to bind to midguts irrelevant. In the few flies that did remain infected, other refractory barriers appeared to exist, such as the parasites lacking the ability to migrate to the anterior midgut, and differentiation to metacyclic promastigotes was not observed.

In contrast to *P.* papatasi, *Lu. longipalpis* supports the development of every species of *Leishmania* tested so far. A natural vector of *L. infantum* in South America, it was surprising to observe an almost complete lack of binding of this parasite when in mixed populations with *L. major* or *L. tropica*. Of course, neither of these Old World parasites are encountered by *Lu. longipalpis* in its natural range, but both bound very strongly to the fly's midgut *in vitro* and effectively out-competed *L. infantum* for binding sites. The nature of the competition effect is unknown, but two possible scenarios are: the parasites are binding to the same receptors, with *L. major* simply having a higher affinity for the receptor; or each species is binding to distinct receptors, but the binding of *L. major* is much stronger and steric hindrance is preventing *L. infantum* from accessing their receptors.

The results with *P. sergenti* also contradict those reported by Kamhawi *et al.* [20] who observed a much lower binding of *L. major* to *P. sergenti* guts in comparison to *L. tropica*. It is possible these apparently conflicting results may have arisen from differences in the experimental design of the binding assays. Two major differences that may account for the higher binding observed here are incubation in different media at different pH and a longer incubation time. Because gut tissue is unstable in PBS after only 10 minutes incubation [20], we decided to use GIM since it is more physiologically similar to the conditions found inside the fly gut and it causes no noticeable changes in the tissue stability for at least 4 hours. The increased health of the gut tissue may alone be enough to account for the differences between the results, but the increase in incubation times from 10 minutes to the 45 minutes used here may also have contributed to a higher observed attachment.

In conclusion, the results of this study confirm that the ability of the *Leishmania* parasite to bind to the midgut epithelium is important and probably essential to transmission. In assessing this interaction it is very important to use the correct life-cycle stages, otherwise erroneous conclusions may be drawn. *In vitro* cultures usually contain a mixture of stages, but can easily lack nectomonad and/or leptomonad promastigotes, depending on culture methodology and time of usage with respect to growth phase (early/mid/late exponential or stationary phase). Such populations will, therefore, lack some or all of the capacity to bind to sand fly midguts. However, whilst binding is necessary it is clearly not sufficient to explain vector specificity and other factors must play a role in limiting the development of parasites in certain vectors. For example, the binding of inappropriate species may not be sufficiently strong in vivo to withstand midgut peristalsis. Conversely, additional factors beyond physical retention may permit the persistence of appropriate parasites in their vectors, and there is the likelihood that strain variation in binding affinity will contribute to observed vector-parasite competence in the field. Other parasite factors important for transmission are the ability of parasites to damage the stomodeal valve [36], [37] and create a blocked fly through secretion of promastigote secretory gel (PSG), a prerequisite for transmission by regurgitation [4], [32]. The fine tuning of parasite development to the digestive physiology of the sand fly is also likely to be one factor, as failure to escape from the peritrophic matrix in time would prevent establishment whether or not a parasite is capable of binding to the midgut. Sand fly factors include the timing and mixture of digestive enzymes produced, trypsin being one known example that exerts an antiparasitic effect [36], [38], [39], as well as the almost completely unknown role of the sand fly immune response in limiting or preventing parasite development [40].

Beyond these it is clear that additional factors affect the *Leishmania* species transmitted by particular sand flies in nature, including the feeding habits of the flies and prevalence of reservoir hosts. From the molecular to the ecological these are all important issues to understand individually and collectively when confronted with the changing epidemiology of leishmaniasis and new or re-emerging foci of disease.
